# Cardiac MRI in Immune Checkpoint Inhibitor Associated Pericarditis

**DOI:** 10.1002/ccr3.4926

**Published:** 2021-10-13

**Authors:** Can Öztürk, Julian A. Luetkens

**Affiliations:** ^1^ Department of Cardiology Heart Center University Hospital Bonn Bonn Germany; ^2^ Department of Diagnostic and Interventional Radiology University Hospital Bonn Bonn Germany; ^3^ Quantitative Imaging Lab Bonn (QILaB) Bonn Germany

**Keywords:** acute pericarditis, cardiac magnetic resonance imaging, immune checkpoint inhibitor, PD‐1 inhibitor

## Abstract

Checkpoint inhibitors are novel and promising anticancer agents. However, acute pericarditis is the second most common chemotherapy‐associated cardiotoxicity and associated with high mortality up to 21%. Cardiac MRI offers a one‐stop‐shop cardiac analysis to precisely detect chemotherapy‐associated cardiotoxicity without nephrotoxic contrast dye and ionizing radiation.

## Graphical abstract

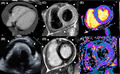

Pericardial effusion was determined in staging computed tomography as an incidental manifestation of acute PD1‐inhibitor‐induced pericarditis. Cardiac MRI showed an extensive contrast enhancement of the thickened pericardium without hinting of myocardial inflammation. Novel mapping techniques offer a promising imaging tool for the adequate detection of chemotherapy‐associated cardiotoxicities.

A 61‐year‐old male patient with stable stage IVB non‐small cell lung adenocarcinoma was recently treated with maintenance chemotherapy consisting of pembrolizumab and pemetrexed for 4 months. Staging computed tomography revealed a circular pericardial effusion (Figure 1, Panel A). In addition, echocardiography confirmed a massive circular PE without signs of hemodynamic impairment (Figure 1, Panel B).

**FIGURE 1:**
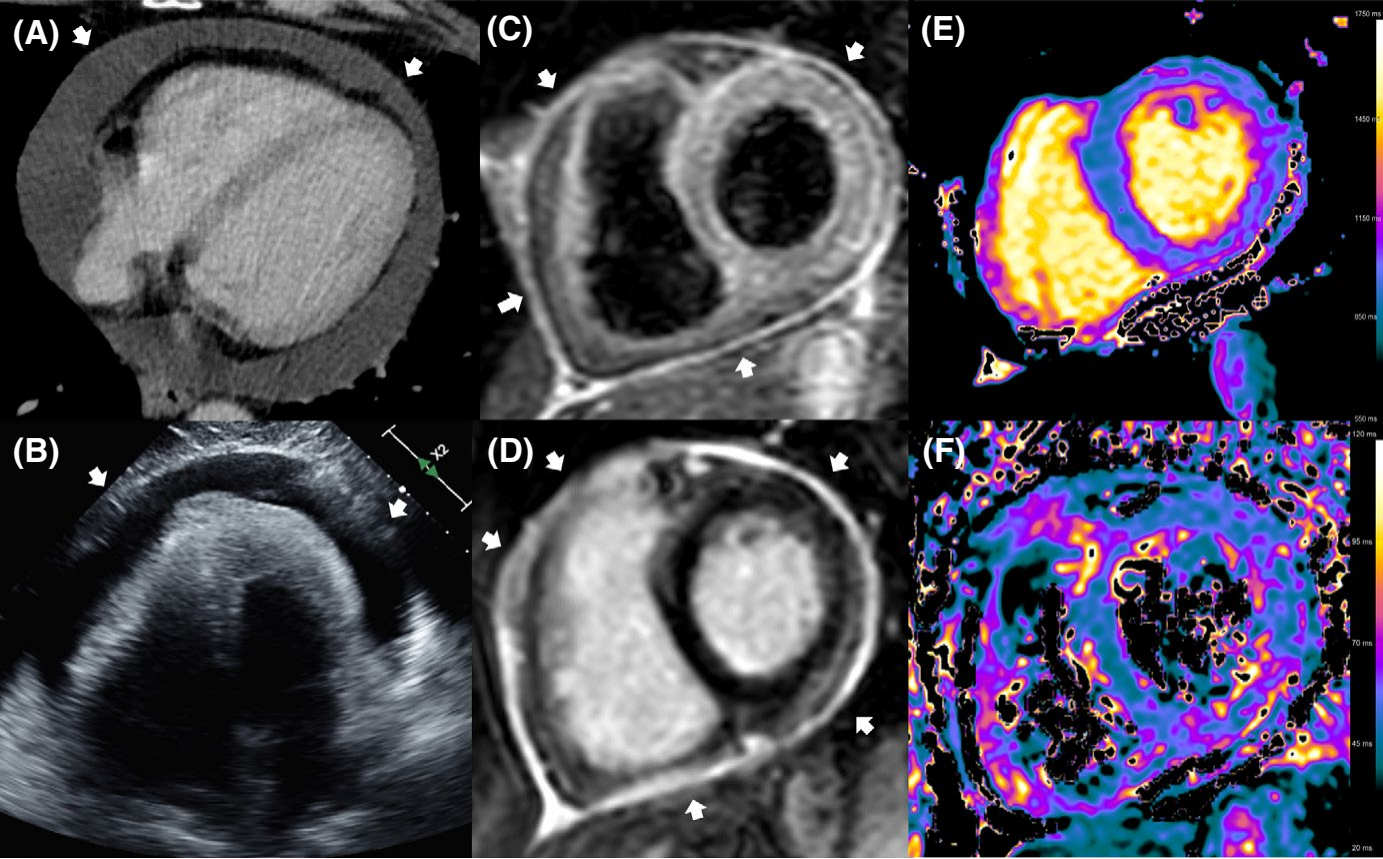
Panel A: Horizontal long‐axis view on an axial slice from cardiac computed tomography with a circular pericardial effusion (arrow). Panel B: Apical four‐chamber view from transthoracic echocardiography with huge circular pericardial effusion (arrow). The end‐diastolic diameter in front of the right ventricle is 28 mm. Panel C: Short‐axis black‐blood T2‐weighted view from cardiac magnetic resonance imaging (MRI) with pronounced pericardial edema (arrow). Panel D: Short‐axis T1‐weighted delayed contrast view from cardiac MRI presents a circular pericardial enhancement (arrow) without any myocardial texture changes (star). Panel E: Representative image of short‐axis native T1 mapping (identifying myocardial fibrosis) from cardiac MRI with normal myocardial texture. Global T1 relation time of 930 ms (center specific cut‐off value for acute myocarditis: >1000 ms). Color scale bar on the right‐hand side. Panel F: Representative image of short‐axis native T2 mapping (identifying myocardial inflammation) from cardiac MRI with normal myocardial texture. Global T2 relaxation time of 54 ms (center specific cut‐off value for acute myocarditis: 55.9 ms). Color scale bar on the right‐hand side

Cardiac MRI showed a marked pericardial hyperintensity in T2‐weighted sequences as a sign of active inflammation (Figure 1, Panel C and F). Moreover, late gadolinium enhancement (LGE) images showed extensive LGE of the thickened pericardium as a sign for acute pericarditis (Figure 1, Panel D). Global T1 and T2 relaxation times and signal intensity ratio were regular (Figure 1, Panel E and F). Histopathologic work‐up of the pericardial fluid (600 ml) found no malignant cells but erythrocytes, reactive mesothelial cells, and acute inflammatory cells. Blood tests ruled out an infection.

Considering clinical suspicion of therapy‐associated pericarditis—most likely PD‐1 inhibitor‐associated—a high‐dose cortisone therapy was started, and the anticancer therapy regime was changed. Control cardiac MRI after 4 weeks showed a dissolved pericardial effusion with decreased pericardial LGE.

This case emphasizes the importance of cardiac MRI in the diagnostic work‐up of patients with suspected acute PD‐1 inhibitor‐associated pericarditis and shows its significance for follow‐up.^1^, ^2^

## CONFLICT OF INTEREST

The authors have no relevant financial or non‐financial interests to disclose.

## AUTHOR CONTRIBUTIONS

CO: involved in writing, image editing and submission; JL: involved in imaging, image editing, and supervision.

## ETHICAL APPROVAL

This case report was in accordance with the Declaration of Helsinki. The patient signed written informed consent for using clinical data for research purposes.

## CONSENT

Published with written consent of the patient.

## Data Availability

Data sharing not applicable to this article as no datasets were generated or analysed during the current study.
